# Exercise Protects Against Olanzapine-Induced Hyperglycemia in Male C57BL/6J Mice

**DOI:** 10.1038/s41598-018-19260-x

**Published:** 2018-01-15

**Authors:** Laura N. Castellani, Willem T. Peppler, Paula M. Miotto, Natasha Bush, David C. Wright

**Affiliations:** Department of Human Health and Nutritional Sciences, University of Guelph, Guelph, Ontario Canada

## Abstract

Olanzapine is a widely prescribed antipsychotic drug. While effective in reducing psychoses, treatment with olanzapine causes rapid increases in blood glucose. We wanted to determine if a single bout of exercise, immediately prior to treatment, would attenuate the olanzapine-induced rise in blood glucose and if this occurred in an IL-6 dependent manner. We found that exhaustive, but not moderate exercise, immediately prior to treatment, prevented olanzapine-induced hyperglycemia and this occurred in parallel with increases in serum IL-6. To determine if IL-6 was involved in the mechanisms through which exhaustive exercise protected against olanzapine-induced hyperglycemia several additional experiments were completed. Treatment with IL-6 (3 ng/g bw, IP) alone did not protect against olanzapine-induced increases in blood glucose. The protective effects of exhaustive exercise against olanzapine-induced increases in blood glucose were intact in whole body IL-6 knockout mice. Similarly, treating mice with an IL-6 neutralizing antibody prior to exhaustive exercise did not negate the protective effect of exercise against olanzapine-induced hyperglycemia. Our findings provide evidence that a single bout of exhaustive exercise protects against acute olanzapine-induced hyperglycemia and that IL-6 is neither sufficient, nor required for exercise to protect against increases in blood glucose with olanzapine treatment.

## Introduction

The use of Second-Generation Antipsychotics (SGAs) such as olanzapine has increased dramatically in recent years^1^. Though traditionally used to manage symptoms of schizophrenia and related illnesses, olanzapine has more recently been prescribed for several off-label conditions including anxiety, chemotherapy-induced nausea, and sleep disorders^1–4^. Despite its growing use, several reports have noted severe metabolic side effects associated with olanzapine^5–7^. Chronic treatment with olanzapine causes marked weight gain and leads to the development of insulin resistance, hyperglycemia and Type 2 Diabetes^7–9^. Though weight gain is closely associated with impaired glucose homeostasis^10^, direct diabetogenic effects of olanzapine have also been observed^11,12^. For example, acute treatment with olanzapine has been shown to induce a rapid rise in blood glucose, independent of changes in body weight, suggesting a direct effect of the drug on glucose homeostasis^12–17^. Though the mechanisms of olanzapine-induced hyperglycemia are not completely understood it is thought to involve increased hepatic glucose output^12^, impaired insulin secretion^18–20^ and reductions in insulin sensitivity^13,16,21^.

Given the impairment in glucose metabolism, combination therapy with glucose lowering drugs would appear to be a logical approach to mitigate the metabolic side effects of olanzapine. Indeed, pairing olanzapine with either metformin or rosiglitazone attenuates the rise in blood glucose^22^. Unfortunately many of these glucose-lowering agents possess undesirable side effects. For example, metformin only partially reverses olanzapine-induced hyperglycemia^23^ and has been reported to worsen psychoses^24^. Similarly, though thiazolidinediones such as rosiglitazone dampen olanzapine-induced perturbations in glucose metabolism^22^ they are associated with weight gain, a worsening of coronary heart disease and increased risk of heart attack^25^. These outcomes highlight the need for alternative approaches to reduce the metabolic side effects of olanzapine.

It has been known for decades that exercise has marked effects on glucose metabolism such as increasing insulin-independent skeletal muscle glucose uptake^26^ and enhancing insulin sensitivity^27^. A more recent investigation has provided evidence that exercise also enhances glucose stimulated insulin secretion^28^. Much work has centered on identifying signaling factors contributing to the beneficial effects of exercise on glucose metabolism. One candidate that has received considerable attention is interleukin 6 (IL-6). IL-6 is secreted from skeletal muscle during exercise in an intensity dependent manner^29^ and some have suggested a role for this cytokine in mediating exercise-induced increases in insulin sensitivity^30,31^ and glucose-stimulated insulin secretion^28^.

Given the pleiotropic effects of exercise on glucose metabolism, it is surprising that the effects of exercise on acute SGA-induced hyperglycemia have never been explored. Within this context, the purpose of the present investigation was to determine if a prior bout of exercise would protect against olanzapine-induced increases in blood glucose. Further, we aimed to assess the role of IL-6 with respect to exercise, as a mechanism to offset olanzapine-induced hyperglycemia. We hypothesized that exercise would blunt SGA-induced hyperglycemia through a mechanism involving IL-6.

## Results

### A single session of exhaustive exercise protects against olanzapine-induced hyperglycemia

**Figure 1:**
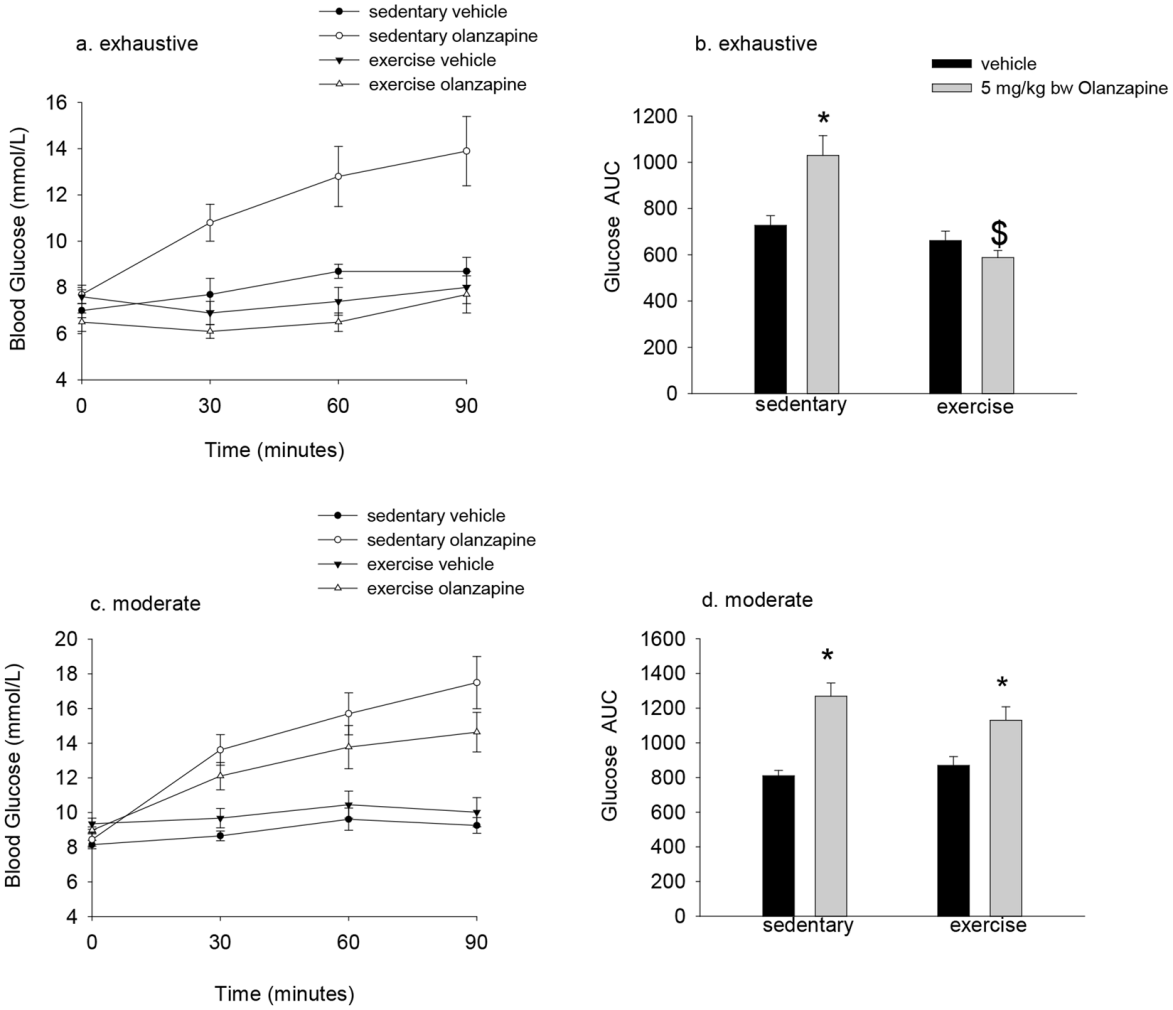
Prior exhaustive exercise prevents olanzapine-induced increases in blood glucose. Mice ran on a motorized treadmill to exhaustion (**a**,**b**) or 75 minutes of moderate (15 m/min) exercise (**c**,**d**) prior to treatment with olanzapine (5 mg/kg bw) or vehicle. Blood glucose was measured prior to and 30, 60 and 90 minutes following treatment (**a**,**c**) and the glucose area under the curve (AUC) calculated (**b**,**d**). Data are presented as means ± SEM for 6–12 mice/group. Groups were compared by 2-way ANOVA. There was a significant interaction (p = 0.007) between exhaustive exercise and olanzapine such that olanzapine increased the blood glucose AUC in sedentary mice (*) while the glucose AUC was different ($) between sedentary and exercised mice treated with olanzapine. *Main effect of olanzapine for increasing blood glucose in the moderate exercise experiment.

We first wanted to determine if a single bout of prior exercise would protect against olanzapine-induced increases in blood glucose. We tested this using both exhaustive and moderate exercise protocols. Exhaustive exercise consisted of a graded exercise protocol (12 m/min, 20° incline to start with the speed increased 1 m/min at 2, 5, 10 and every subsequent 10 minutes) until exhaustion. Mice ran for 77 ± 4 minutes during this exercise protocol. In sedentary mice, olanzapine treatment resulted in a significant (p = 0.007) increase in the blood glucose area under the curve (AUC) and this was completely prevented by a prior bout of exhaustive exercise (p < 0.001 sedentary compared to exercised mice treated with olanzapine) (Fig. 1a,b). We next examined if a more clinically relevant, moderate exercise would also confer protection against olanzapine-induced increases in blood glucose. Mice ran for approximately the same duration (75 minutes) as the exhaustive exercise protocol, but at 15 m/min and a 5° incline, a speed and grade that elicits ~75% of VO_2_ max in male C57BL6 mice^32^. In this experiment olanzapine increased the blood glucose AUC (main effect p < 0.001) in both sedentary and exercised mice (Fig. 1c,d).

### Serum insulin is increased following olanzapine treatment in mice exercised to exhaustion

**Figure 2:**
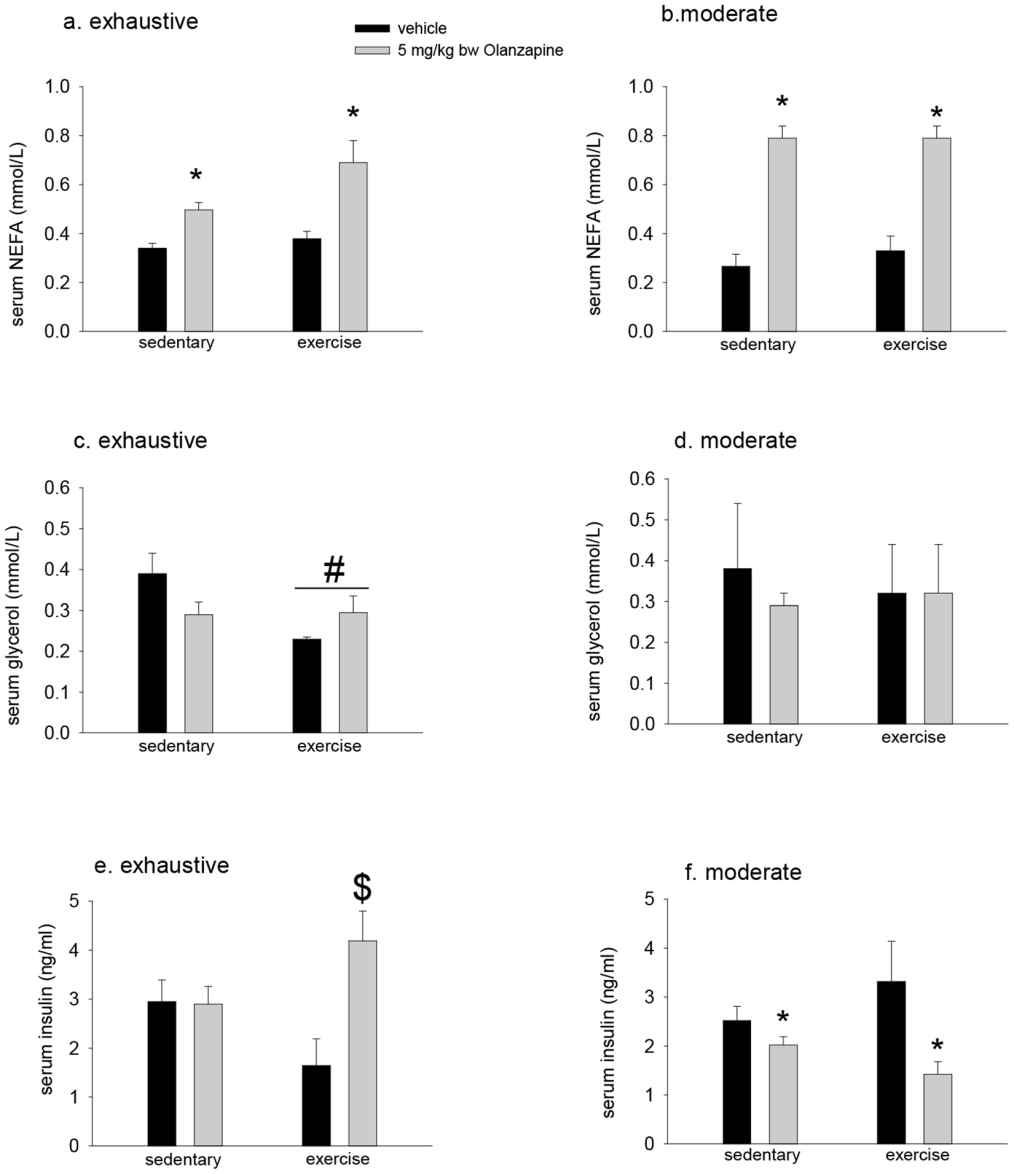
Prior exhaustive exercise increases serum insulin in olanzapine treated mice. Mice ran on a motorized treadmill to exhaustion (**a**,**c**,**e**) or an equivalent duration of moderate (15 m/min) exercise (**b**,**d**,**f**) prior to treatment with olanzapine (5 mg/kg bw) or vehicle. 90 minutes post treatment serum was collected and for measurement of non-esterified fatty acids (NEFA) (**a**,**b**), glycerol (**c**,**d**) and insulin (**e**,**f**). Data are presented as means ± SEM for 6–12 mice/group. Groups were compared by 2-way ANOVA. There was significant (p = 0.003) interaction between exhaustive exercise and olanzapine such that serum insulin levels were increased in previously exercised mice treated with olanzapine compared to vehicle ($). ^#^Main effect of exercise, *main effect of olanzapine.

We next wanted to determine if the divergent effects of the exhaustive and moderate exercise protocols in protecting against olanzapine-induced hyperglycemia could be explained, at least in part, by differences in circulating markers of lipid and carbohydrate metabolism. As shown in Fig. 2 there was a main effect of olanzapine (p < 0.001) for increasing serum NEFA concentrations 90 minutes after olanzapine treatment. This effect was not altered by previous exhaustive (Fig. 2a) or moderate (Fig. 2b) exercise. In contrast to NEFA, there was a main effect of exhaustive (p = 0.045) (Fig. 2c) but not moderate exercise (Fig. 2d) in reducing serum glycerol concentrations 90 minutes following treatment with vehicle or olanzapine. There was a trend (p = 0.064) for serum insulin levels to be reduced in vehicle treated mice exercised to exhaustion compared to sedentary mice given vehicle. Serum insulin levels were significantly increased (p = 0.004) in mice that had exercised to exhaustion and were treated with olanzapine compared to exercised vehicle treated mice (Fig. 2e). In contrast to the exhaustive exercise experiment there was a main effect of olanzapine (p = 0.016) to reduce serum insulin concentrations 90 minutes post treatment in the moderate exercise experiment (Fig. 2f).

### Exhaustive exercise leads to increases in phosphorylated AMPK, IL-6 and GLP-1

**Figure 3:**
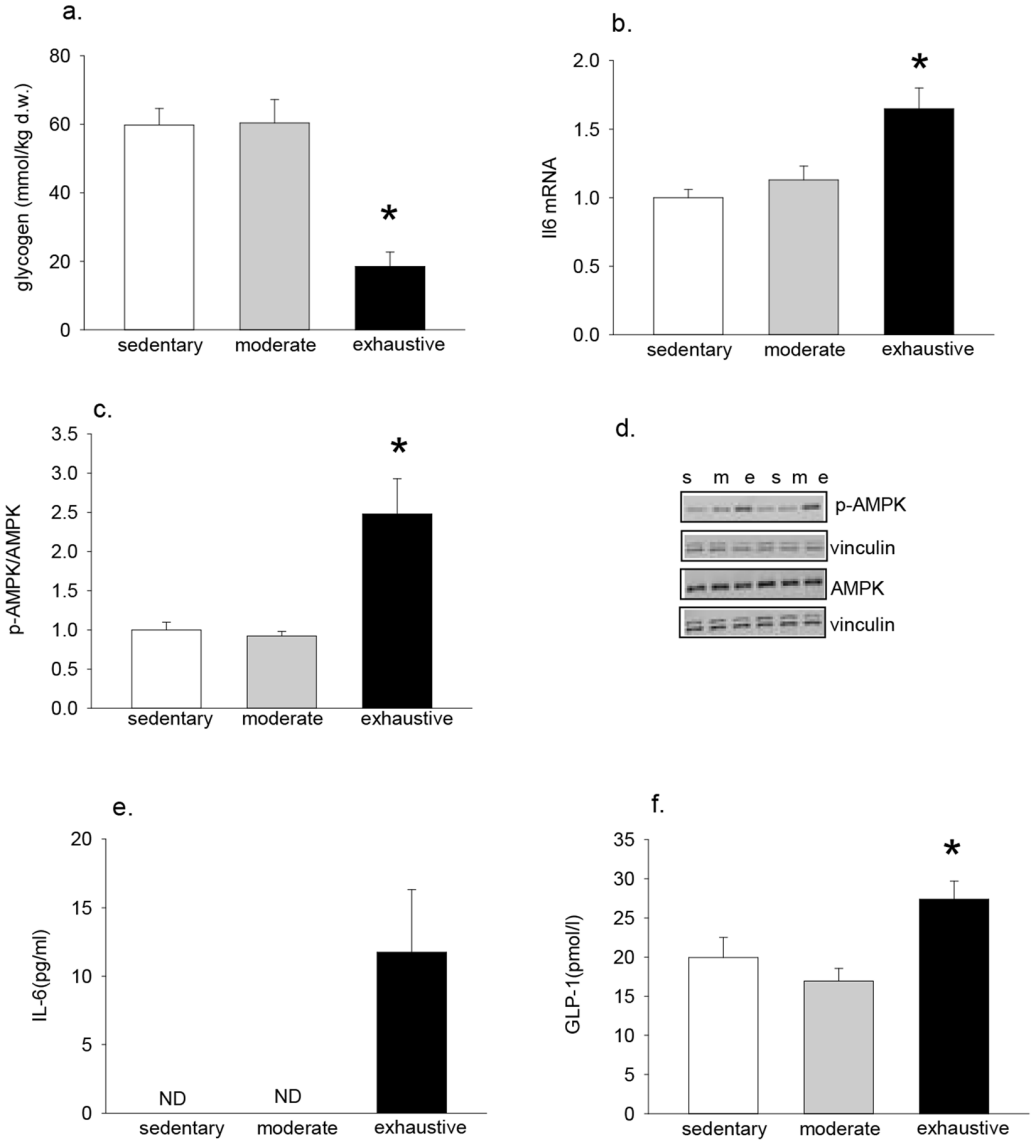
Exhaustive exercise increases IL-6 and GLP-1. Mice ran on a motorized treadmill to exhaustion or an equivalent duration of moderate (15 m/min) exercise. Immediately following exercise tissues were harvested and later analyzed for muscle glycogen (**a**), muscle IL-6 mRNA (**b**), p-AMPK (**c**,**d**) serum IL-6 (**e**) and serum GLP-1 (**e**). Data are presented as means ± SEM for 5–6 mice/group. Groups were compared by one-way ANOVA. *Different than sedentary and moderate exercise. N.D. = not detectable. s = sedentary, m = moderate exercise, e = exhaustive exercise.

As exhaustive exercise protected against olanzapine-induced increases in blood glucose, and this was paralleled with increases in serum insulin concentrations, we wanted to determine if this was associated with increases in IL-6, an exercise-induced myokine linked to increases in insulin secretion^28^. Skeletal muscle is thought to be the primary source of exercise-induced increases in circulating IL-6^33^ with the induction of IL-6 occurring to a greater extent in conditions of reduced muscle glycogen^34^. Vastus glycogen concentrations were significantly reduced immediately following exhaustive exercise compared to moderate exercise (p < 0.001) and sedentary (p < 0.001) conditions (Fig. 3a). Similarly IL-6 mRNA expression was increased immediately following exhaustive exercise compared to the sedentary (p < 0.001) and moderate (p = 0.003) exercise groups (Fig. 3b). The phosphorylation of the cellular energy sensor 5’AMP activated protein kinase (AMPK), was increased in vastus muscle following exhaustive, but not moderate exercise (Fig. 3c,d). Serum IL-6 concentrations were below the detectable limit in sedentary mice and following moderate exercise whereas IL-6 levels were ~12 pg/ml immediately following exhaustive exercise (Fig. 3e). As IL-6 has been linked to exercise-induced increases in GLP-1^28^ we next sought to determine if exercise led to increases in circulating concentrations of this hormone. In line with our IL-6 data, serum GLP-1 was increased immediately following exhaustive exercise compared to the moderate exercise (p = 0.003) and sedentary groups (p = 0.021) (Fig. 3f).

### IL-6 is not sufficient to protect against olanzapine-induced hyperglycemia

**Figure 4:**
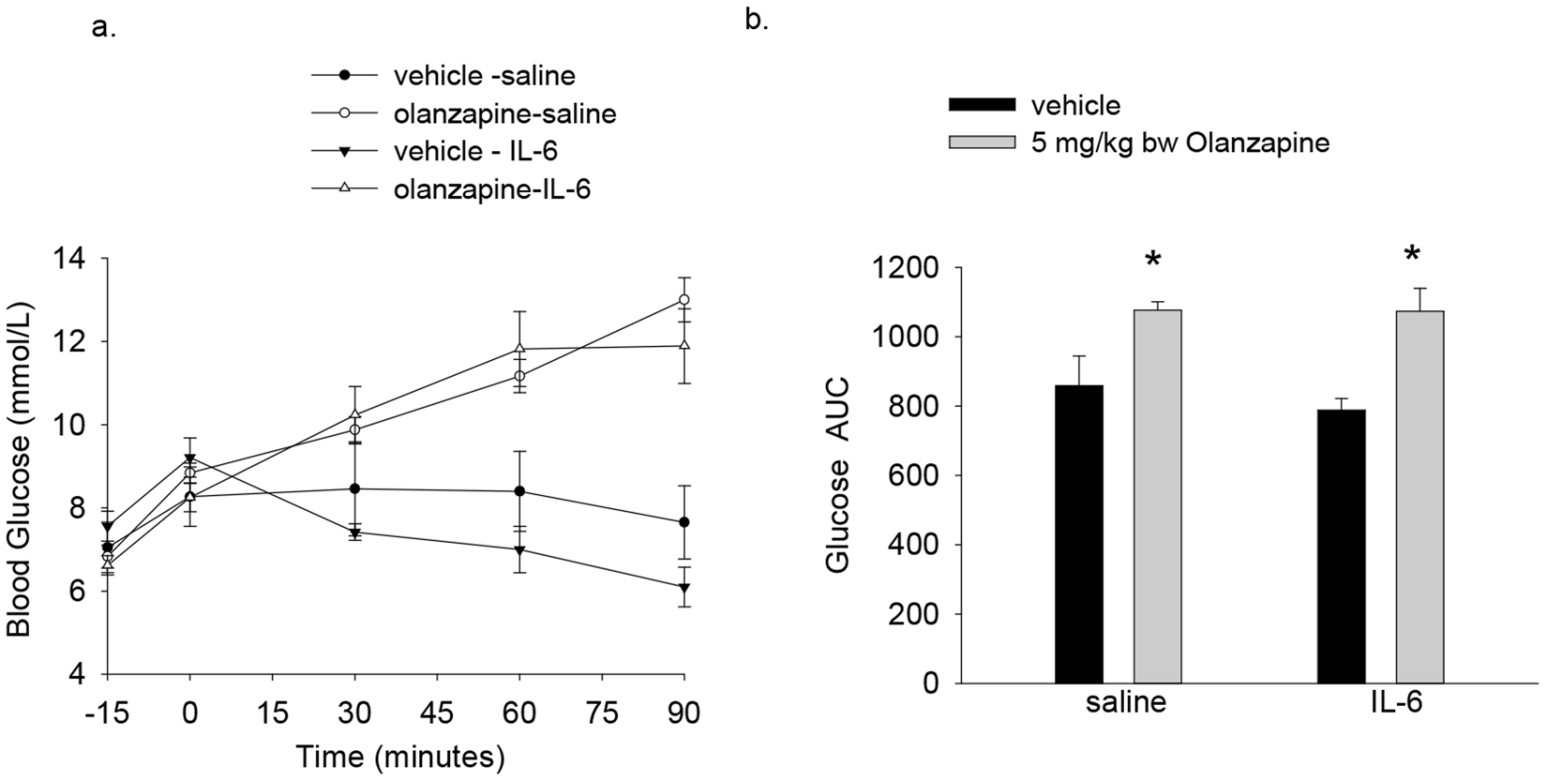
IL-6 is not sufficient to protect against olanzapine-induced hyperglycemia. C57BL/6 J Mice were injected with murine recombinant IL-6 (3 ng/g bw) or an equivalent volume of saline and 15 minutes later treated with vehicle or olanzapine (5 mg/kg bw). Blood glucose was measured before IL-6 injections (−15), 15 minutes following (0) and 30, 60 and 90 minutes after olanzapine or vehicle treatment (**a**) and the area under the curve (AUC) calculated (**b**). Data are presented as means ± SEM for 6–10 mice/group. Groups were compared by a 2 way ANOVA (**c**). *Main effect of olanzapine. Please note, the glucose AUC was calculated from 0–90 minutes.

Having shown that exhaustive exercise protects against olanzapine-induced hyperglycemia in parallel with increases in IL-6 we wanted to examine if increases in IL-6 were sufficient to protect against increases in blood glucose following olanzapine treatment. To address this question mice were injected with recombinant murine IL-6 (3 ng/g bw) or an equivalent volume of saline and 15 minutes later mice were treated with olanzapine and blood glucose measured over the subsequent 90 minutes. Fifteen minutes post-IL-6 treatment serum IL-6 concentrations were significantly (P < 0.05) increased compared to vehicle treated control animals (vehicle 29 ± 13, IL-6 267 ± 76 pg/ml, n = 5–9/group). Olanzapine increased (main effect olanzapine p = 0.008) the blood glucose AUC in mice treated with either saline or IL-6 15 minutes prior to olanzapine treatment (Fig. 4a,b).

It is possible that the duration of IL-6 exposure was not long enough to confer a protective effect against olanzapine-induced excursions in blood glucose. We therefore repeated the above experiments and challenged mice with olanzapine 60 minutes following treatment with saline or IL-6. As with our initial experiment IL-6 treatment did not attenuate olanzapine-induced hyperglycemia (saline + olanzapine 1381 ± 94 glucose AUC, IL-6 + olanzapine 1403 ± 120 glucose AUC, n = 8/group).

### IL-6 is not required for exhaustive exercise to protect against olanzapine-induced hyperglycemia

**Figure 5:**
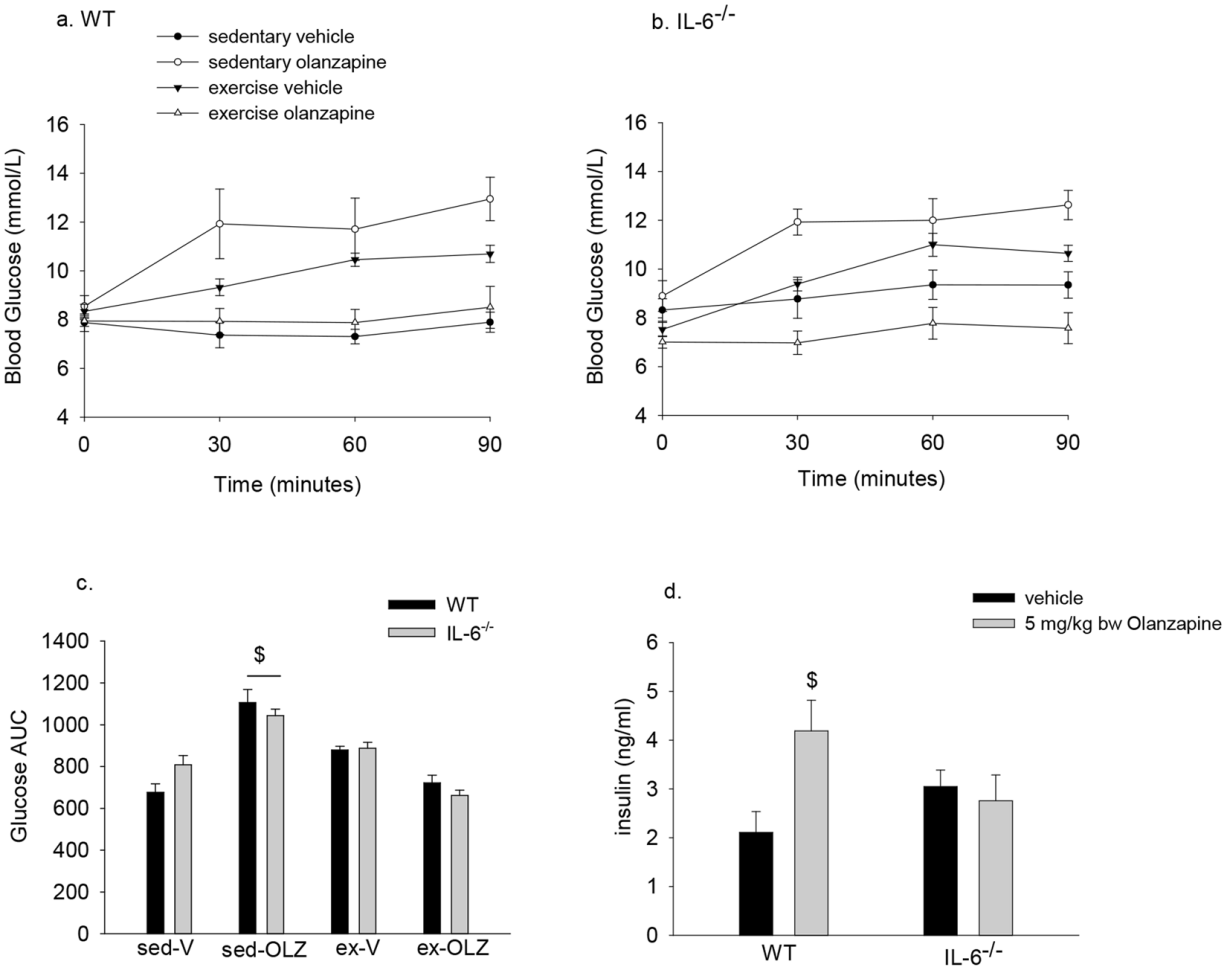
The protective effects of exercise against olanzapine-induced hyperglycemia are intact in IL-6^−/−^ mice. WT or IL-6^−/−^ mice underwent the exhaustive exercise protocol and then immediately were treated with olanzapine (5 mg/kg bw) or vehicle. Blood glucose was measured prior to and 30, 60 and 90 minutes post treatment (**a**,**b**) and the glucose area under the curve (AUC) calculated (**c**). Serum insulin concentrations were determined 90 minutes following olanzapine treatment in exercised WT and IL-6^−/−^ mice (**d**). Data are presented as means ± SEM for 6–10 mice/group. Groups were compared by a 3 (**c**) or 2 (**d**) way ANOVA. ^$^Significant interaction (p < 0.001) in (**c**) such that sed-OLZ was different than sed-V and ex-OLZ. ^$^Significant interaction (p = 0.003) such that WT olanzapine is different that WT vehicle in d. sed-V = sedentary vehicle, sed-olz = sedentary olanzapine, ex–V = exercise vehicle, ex-OLZ = exercise olanzapine.

While IL-6 alone, at least at the dosage used, was not sufficient to protect against olanzapine-induced hyperglycemia this does not discount a potential role for IL-6 in mediating the protective effects of exhaustive exercise. To address this question WT or whole body IL-6^−/−^ mice were exercised to exhaustion and then treated with olanzapine or vehicle and blood glucose measured over the subsequent 90 minutes. Olanzapine treatment significantly (p = 0.007) increased blood glucose AUC in both genotypes and this was prevented by prior exercise (sedentary olanzapine, compared to exercise olanzapine p < 0.001) (Fig. 5a–c). Despite the lack of a genotype effect with exercise in protecting against olanzapine-induced hyperglycemia there were differences between WT and IL-6^−/−^ mice with regards to serum insulin concentrations. Following exhaustive exercise in WT mice treated with olanzapine there was a significant increase (p = 0.011) in serum insulin compared to mice that had exercised and were given vehicle (Fig. 5d). This increase with exhaustive exercise was absent in IL-6^−/−^ mice.

As the lifelong whole body deletion of IL-6 could lead to adaptive changes that could impact the responses to exercise and/or olanzapine, we thought it necessary to confirm our findings using a different model. To this end, we repeated the exhaustive exercise experiments in the presence, or absence, of acute treatment with an IL-6 neutralizing antibody (0.5 mg/mouse). This dose of neutralizing antibody has previously been shown to block fasting-induced increases in lipolysis in mice^35^ and reduces IL-6 mediated increases in serum amyloid A^28^. Notably, though olanzapine increased blood glucose one-hour post IgG control or IL-6 neutralizing antibody in sedentary mice, exhaustive exercise protected against olanzapine-induced increases in blood glucose in mice treated with either IL-6 neutralizing antibody or control IgG.

**Figure 6:**
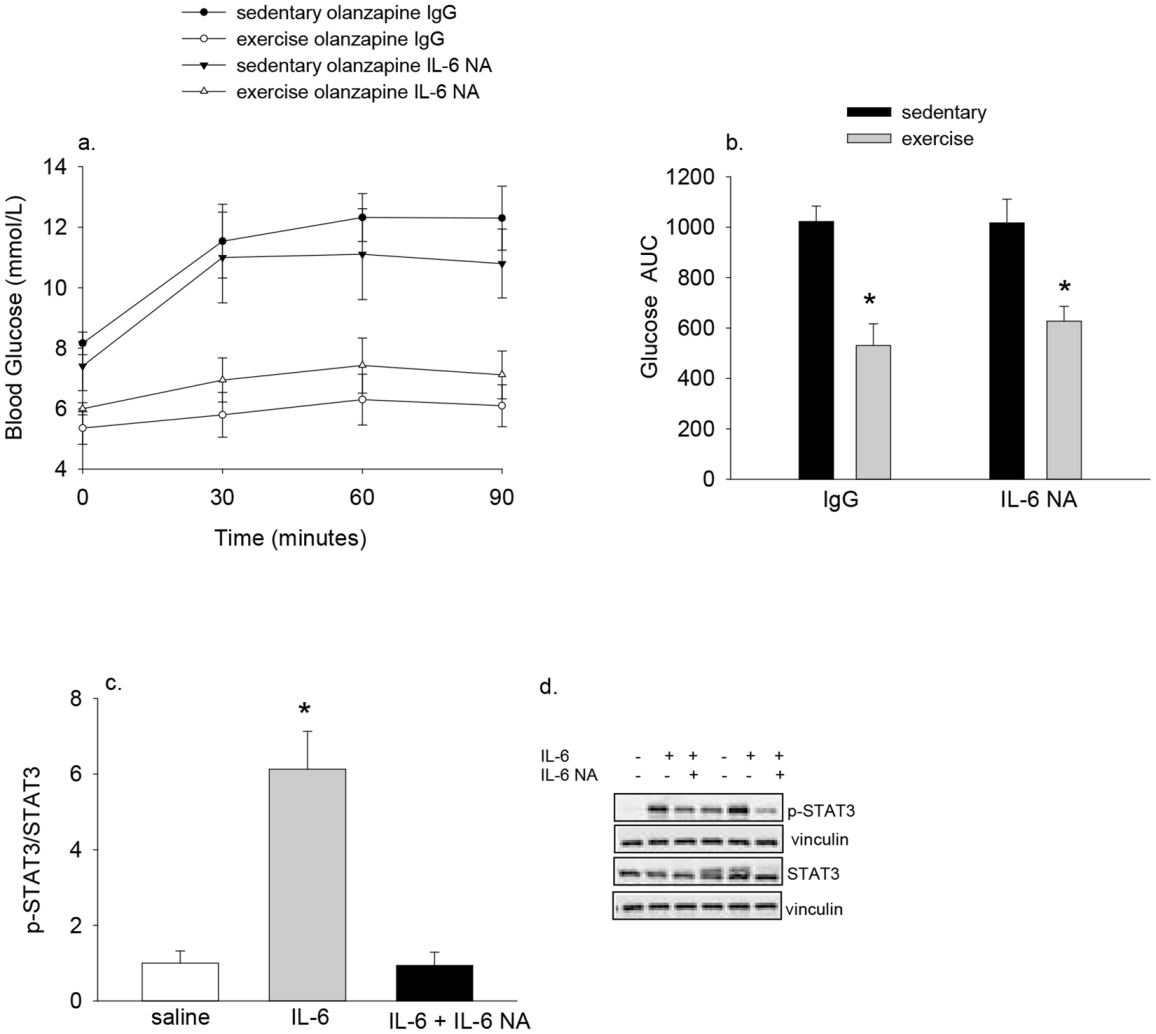
The protective effects of exercise against olanzapine-induced hyperglycemia are intact in mice treated with an IL-6 neutralizing antibody. Male C57BL6/J mice were injected with an IL-6 neutralizing antibody (IL-6 NA, 0.5 mg/mouse) or an equivalent amount of IgG control antibody. 60 minutes later mice performed the exhaustive exercise protocol, or remained sedentary, and were treated with olanzapine (5 mg/kg bw) immediately following exercise. Blood glucose was measured immediately prior to and 30, 60 and 90 minutes post olanzapine (**a**) and the glucose AUC determined (**b**). To ensure that the IL-6 NA was effective in attenuating IL-6 signaling, mice were treated with the IL-6 NA (0.5 mg/mouse) and 60 minutes later injected with IL-6 (3 ng/g bw, IP). 15 minutes post IL-6 injection livers were harvested and analyzed for changes in the phosphorylation of STAT3 (**c,d**). Data are presented as means ± SEM for 6–10 mice/group. Data were analyzed by 2 way ANOVA in b and one way ANOVA in C. *Main effect of exercise in b, IL-6 different than the other two groups in (**c**). Representative Western blots are shown in d.

To confirm the effectiveness of the IL-6 neutralizing antibody we assessed whether it could attenuate IL-6-induced increases in the phosphorylation of STAT3 (Signal Transducer and Activator of Transcription3). While we surprisingly were unable to detect IL-6 induced increases in STAT3 phosphorylation in muscle (data not shown), pre-treatment with IL-6 neutralizing antibody completely prevented IL-6 induced increases in liver STAT3 phosphorylation (Fig. 6c,d).

## Discussion

SGAs are widely used in the treatment of schizophrenia^1^ and several other off-label conditions^1–4^. Although effective in reducing psychosis these drugs have noted metabolic side effects including rapid increases in blood glucose^16^. The acute metabolic effects are particularly problematic as they persist with long-term usage^36^. As the majority of pharmaceutical treatment options for the metabolic side effects of SGAs have limitations, the identification of alternative approaches is needed. Recent work from Boyda *et al*.^37^ found that regularly performed wheel running protected against SGA-induced weight gain and glucose intolerance in rats. Similarly, clinical investigations have demonstrated that exercise in combination with alterations in diet leads to reductions in adiposity^38^ and fasting blood glucose concentrations^39^ in individuals with severe mental illness treated with SGAs. While these studies suggest that exercise can be used to offset the chronic metabolic side effects of SGAs, it is difficult to determine if exercise has direct effects on protecting against SGA-induced perturbations in glucose homeostasis, or if it is secondary to a prevention of weight gain/the induction of weight loss. In the current study we have been the first to show that a single bout of exhaustive exercise prior to drug treatment, completely attenuated olanzapine-induced increases in blood glucose. These findings, and the work of others^38,39^, highlights beneficial effects of both acute and chronic exercise in protecting against the metabolic side effects of SGAs.

We examined the effects of two different exercise protocols and found that exhaustive, but not moderate exercise protected against olanzapine-induced hyperglycemia. This divergent response to exercise allowed us to examine potential mechanisms that could be mediating the protection against olanzapine-induced increases in blood glucose. With exhaustive, but not moderate exercise, there was an induction of IL-6 mRNA in skeletal muscle and increases in circulating IL-6 concentrations. These findings provide associative evidence that exercise-induced increases in IL-6 could be mediating the protective effects of exhaustive exercise against olanzapine-induced hyperglycemia. However, several lines of evidence argue against this. First, treating mice with IL-6, albeit using a dose that results in serum IL-6 levels higher than that seen following exercise, was not able to protect against olanzapine-induced increases in blood glucose. Although we used the same dose of IL-6 as has been used previously^40,41^, and the relative increase in serum IL-6 was similar (~6–9 fold increase), absolute circulating IL-6 concentrations were higher in the present study than what has previously been reported (~60–140 ng/ml)^40,41^. Additionally, we found that the protection against olanzapine-induced hyperglycemia with prior exhaustive exercise was intact in whole body IL-6^−/−^ mice or in mice that had been treated with an IL-6 neutralizing antibody. These results provide evidence that increases in IL-6 are neither sufficient nor required for the effects of exhaustive exercise in protecting against olanzapine-induced excursions in blood glucose and begs the question as to the specific mechanisms that could be mediating the protective effects of exercise. While speculative, and an area requiring further attention, it is possible that AMPK, a reputed mediator of exercise-induced increases in muscle glucose uptake^42^ and insulin sensitivity^43^, could play a role in mediating the effects of exhaustive exercise against olanzapine-induced hyperglycemia.

Although exhaustive exercise protected against olanzapine-induced hyperglycemia in IL-6^−/−^ mice, we found that exercise-induced increases in serum insulin from olanzapine compared to vehicle treated animals were absent. These findings are consistent with recent work demonstrating linkages between increases in IL-6 and serum insulin concentrations^28,44^. The attenuated rise in serum insulin in IL-6^−/−^ mice is likely not the result of an overall reduced capacity of the pancreas to produce insulin. In support of this, though it should be mentioned that insulin secretion per se was not directly measured, the ability of the beta-3 adrenergic agonist, CL 316, 243 to increase serum insulin levels is intact in IL-6^−/−^ mice^45^.

While serum insulin was not increased in exercised IL-6^−/−^ mice treated with olanzapine, the rise in blood glucose was prevented in these animals providing evidence that protection against olanzapine-induced hyperglycemia with exercise is not mediated by increases in circulating insulin. Additional lines of evidence also demonstrate that increases in serum insulin are not required, or sufficient to protect against acute SGA-induced increases in blood glucose. For example we recently reported that sedentary glucagon receptor knockout mice are protected against olanzapine-induced hyperglycemia in the absence of increases in insulin^46^. Similarly, treating rats with glyburide, a sulfonylurea which increases insulin secretion, was not effective in protecting against SGA-induced glucose intolerance^22^.

Although our findings provide intriguing evidence that exhaustive exercise could be used as a tool to protect against the metabolic side effects of SGAs, caution should be taken when extrapolating this to clinical endpoints as persons with severe mental illness such as schizophrenia engage in significantly less physical activity than the general population^47^. There are a number of underlying symptoms in this patient group that would likely preclude exercise being easily incorporated into treatment regimes. For example, the negative symptoms of schizophrenia which include apathy, amotivation, and social withdrawal^48^ are not altered with SGA treatment and could be one factor that is linked to poor exercise adherence, especially when unsupervised, in individuals with schizophrenia^49^. Another point to consider is that exhaustive exercise was required to protect against the acute effects of olanzapine on blood glucose. It seems unlikely, even under the best of circumstances, that individuals would be willing or able, to do this on a daily basis. With these points in mind, further studies are needed to elucidate the specific mechanisms through which exhaustive exercise protects against SGA-induced hyperglycemia. The findings of such studies could pave the way for the design of targeted approaches to mimic the beneficial effects of exhaustive exercise in protecting against SGA-induced perturbations in glucose homeostasis.

## Methods

### Data Availability

The datasets generated during and/or analyzed during the current study are available from the corresponding author on reasonable request.

### Materials

ELISAs for IL-6 (cat. no. EZMIL6), insulin (cat. no. EZRMI-13K) and GLP-1 (cat. no. EZGLP1–36K) were obtained from Millipore (Billerica, MA, USA). Olanzapine (cat. no. 11937) was purchased from Cayman Chemicals (Cat. No. 11937) (Ann Arbor, MI, USA). During the revision of this manuscript Millipore discontinued the IL-6 ELISA. For the analysis of IL-6 from IL-6 injected mice, we purchased a mouse specific IL-6 ELISA from Sigma (Oakville, Ontario)(cat. No. RAB0308). Mouse IL-6 neutralizing antibodies (cat. No. AB-406-NA) and IgG control (cat. No. AB-108-C) were from R&D Systems (Minneapolis, MN). Recombinant murine IL-6 (cat. No. 216-16) was obtained from Peprotech (Rocky Hill, NJ). Nonesterified free fatty acid (NEFA) kits (NEFA-HR kits), glycerol kits and dimethyl sulfoxide (DMSO) were purchased from Wako Chemicals (Richmond, VA, USA). Primary antibodies against tyr. 705 phosphorylated STAT3 (signal transducer and activator of transcription 3) (cat. No. 9145), STAT3 (cat. No. 4904), thr. 172 phosphorylated AMPK alpha (cat. No. 2535) and total AMPK alpha (cat. No. 2532) were from Cell Signaling (Danvers, MA). Vinculin antibodies (cat. No. 05–386) were from Millipore (Etobicoke, ON). A Freestyle Lite handheld glucometer and blood glucose test strips were purchased from Abbott Diabetes Care Inc. (Alameda, CA, USA). All other reagents were purchased from Sigma Aldrich (St Louis, MO, USA).

### Animals

8-week old male C57BL/6 J mice and IL6^−/−^ (B6.12952-IL6^tmlkopf^/J) mice were purchased from Jackson Laboratory (Bar Harbor, ME, USA) and housed 1 per cage with free access to standard rodent diet (7004- Teklad S-2335 Mouse Breeder Sterilizable Diet; Teklad Diets Harlan Laboratories, Madison WI, USA) and water. Mice were housed on a 12 h:12 h light:dark cycle (lights turned on at 7:00 h and turned off at 19:00 h) at 23 °C. C57BL/6 J mice were used as wild type controls (as suggested by the supplier) as they provided the background used to backcross the IL-6^−/−^^50,51^. The IL-6^−/−^ mice used in these experiments are crossed every 10 generations to prevent genetic drift^51^. All protocols were approved by the University of Guelph Animal Care Committee and followed the guidelines of the Canadian Council on Animal Care.

### Drug Preparation

Olanzapine was first dissolved in DMSO (10 mg/mL) and subsequently prepared in a solution containing 90% sterile saline (0.9% NaCl), 5% DMSO and 5% Kolliphor at a concentration of 0.5 mg/mL. Vehicle solution contained matched proportions of sterile saline, DMSO and Kolliphor without olanzapine. Animals were injected, I.P. with 5 mg/kg body weight as this dose has been previously shown to induce a rise in blood glucose^12^ as well as reflect a clinically relevant dopamine D_2_ receptor occupancy of ~70% in rats^52^.

### Experimental Procedures

#### Acute Exhaustive Exercise

Mice were exercised on a motorized treadmill as previously described^53^. Briefly, mice performed a single session of treadmill running, at an incline of 20° and an initial speed of 12 meters/minute. Speed was increased 1 meter/minute at 2, 5, 10, and every subsequent 10 minutes until mice were exhausted (~75 minutes). Exhaustion was determined as an inability to remain halfway up the treadmill belt 3 consecutive times despite gentle prodding. Age matched mice remained in their cage without access to food for the duration of the exercise protocol. Immediately post-exercise blood glucose was measured by handheld glucometer and mice were injected (I.P.) with 5 mg/kg bw olanzapine or an equivalent amount of vehicle solution. Blood glucose was then measured every 30 minutes, for 90 minutes after which mice were anesthetized with a weight-adjusted injection of sodium pentobarbital (5 mg/100 g body weight; I.P.) and vastus muscle collected and frozen at −80 °C for further analysis. Cardiac blood was collected, and centrifuged at 5,000 G for 10 minutes at 4 °C. Serum was collected and stored at −80 °C until further analysis. A subset of mice were killed immediately post-exercise and serum and muscle collected and frozen at −80 °C.

#### Acute Moderate Intensity Exercise

Male C57BL/6 J mice were exercised using a moderate exercise protocol (~75% of VO_2max_) as described previously^32^. Briefly, mice ran on a motorized treadmill for 75 minutes at a speed of 15 meters per minute and an incline of 5°. Immediately post-exercise mice were treated with a weight-adjusted bolus of olanzapine, or vehicle solution and blood glucose was measured 30, 60, and 90 minutes post-treatment.

#### Recombinant IL-6 injections

Mice were treated with sterile saline or recombinant IL-6 (3 ng/g body weight; i.p. injection). In a subset of mice liver was collected 15-minutes post and total and phosphorylated STAT-3 protein content was measured by Western blotting as we have described in detail previously^32^. 15 or 60 minutes post saline or IL-6 treatment, olanzapine or vehicle solution was administered (5 mg/kg, i.p. injection). Blood glucose was measured 30, 60 and 90 minutes post-olanzapine and area under the curve was calculated.

IL-6 Neutralizing Antibody Experiments: Mice were injected with IL-6 neutralizing antibody (0.5 mg) or an equivalent amount of control IgG. Blood glucose was measured immediately pre, and 60 minutes post antibody injection. 60 minutes post-injection mice underwent the exhaustive exercise protocol (as described above) or remained sedentary and were then immediately treated with olanzapine (5 mg/kg; i.p. injection). Blood glucose was measured 0, 30, 60 and 90 minutes post-olanzapine and area under the curve was calculated.

#### Serum IL-6, insulin, NEFA and glycerol

Serum concentrations of IL-6, insulin and GLP-1 were detected using commercially available enzyme-linked immunosorbent assays (ELISAs), according to manufacturer’s instructions. Samples from each experiment were run on the same plate, in duplicate with an average CV of <10%. Serum NEFA and glycerol concentrations were measured using commercially available kits as previously described in detail^45^.

#### Muscle Glycogen

Glycogen concentrations were determined in vastus muscle as described in detail previously^54^.

#### Western Blotting

Samples were prepared and analyzed for western blotting as previously described^32^. Samples were homogenized (TissueLyser LT, Qiagen) in cell lysis buffer (cat. No. FNN0021 LifeTech), supplemented with phenylmethylsulfonyl fluoride and protease inhibitor cocktail (Sigma-Aldrich). Homogenized samples were centrifuged at 4 °C (10 minutes at 5,000 × G), and the supernatant collected and protein content determined using a bicinchoninic acid assay^55^. Equal amounts of protein were separated on 10% SDS-PAGE gels and transferred onto nitrocellulose membranes using a wet transfer technique (at 200 mA/transfer unit). Membranes were blocked in tris buffered saline/0.1% tween with 5% non-fat dry milk for 1 h then incubated in TBST/5% bovine serum albumin supplemented with the appropriate primary antibody (1:1000 dilution) at 4 °C overnight with gentle agitation. After incubation in primary antibody solution, membranes were rinsed with TBST and incubated with HRP-conjugated secondary antibodies for 1 h at room temperature. Secondary antibodies were diluted in TBST with 1% non-fat dry milk. Signals were detected using enhanced chemiluminesence and were then quantified by densitometry using a FluorChem HD imaging system (Alpha Innotech, Santa Clara, CA). Proteins of interest (total and phosphorylated AMPK and STAT3) were normalized to a within gel loading control (vinculin), the ratio of the phosphorylated to non-phosphorylated protein was calculated and values expressed relative to sedentary or saline conditions.

#### Real Time PCR

Changes in mRNA expression were determined using real time qPCR as previously described by our laboratory^56^. Briefly, RNA was isolated from vastus muscle using an RNeasy kit according to the manufacturer’s instructions (Qiagen) followed by DNase free treatment. Complementary DNA (cDNA) was synthesized from total RNA (1 μg) using dNTP, SuperScript II Reverse Transcriptase, and random primers (ThermoFisher). Real time PCR was completed with TaqMan gene expression assays (ThermoFisher) using a 7500 Fast Real-Time PCR system (Applied Biosystems). The 2^−∆∆CT^ method was used to calculate fold differences in gene expression with Gapdh used as a housekeeping gene^57^.

### Statistical Analysis

Differences between groups were analyzed using either a 1, 2 or 3 way ANOVA, with LSD post-hoc analysis. A relationship was considered significant when P < 0.05. Please note that statistical analysis was completed on the glucose AUC data and not the glucose time course data.

## Electronic supplementary material

Supplementary Information
