# Supplementary material for: Exercise Protects Against Olanzapine-Induced Hyperglycemia in Male C57BL/6J Mice
Source: Sci Rep. 2018 Jan 15;8:772. doi: 10.1038/s41598-018-19260-x (PMC5768692; doi:10.1038/s41598-018-19260-x)

Exercise Protects Against Olanzapine-Induced Hyperglycemia in Male C57BL/6J Mice

Laura N. Castellani[1]; Willem T. Peppler[1]; Paula M. Miotto[1]; Natasha Bush[1]; David C. Wright, PhD[1*]

[1]Department of Human Health and Nutritional Sciences, University of Guelph, Guelph Ontario Canada

Supplementary File: Western blot

Vastus muscle

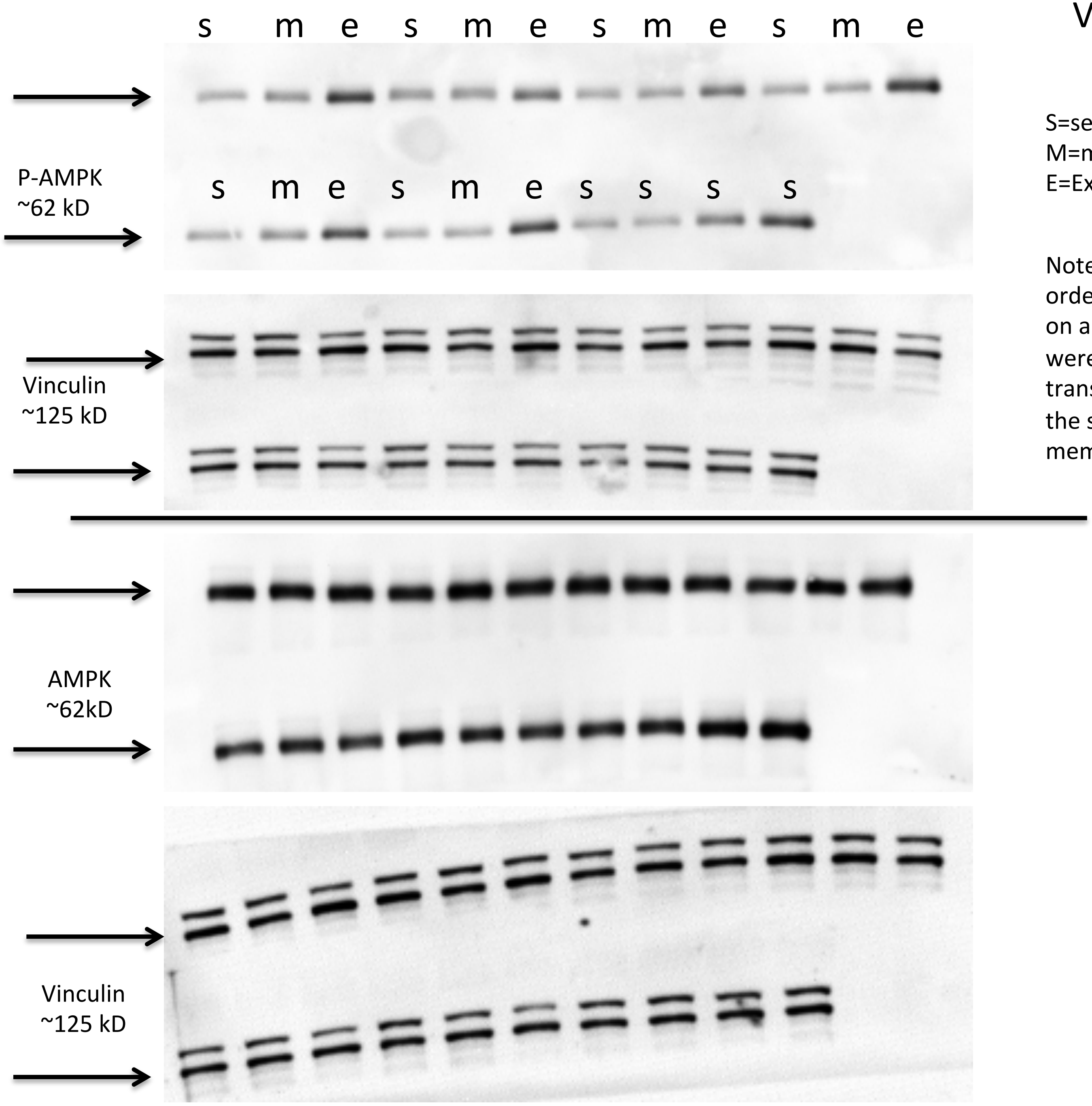

S=sedentary
M=moderate
E=Exhaustive

Note: loading order the same on all gels. Gels were cut and transferred on the same membrane

Liver

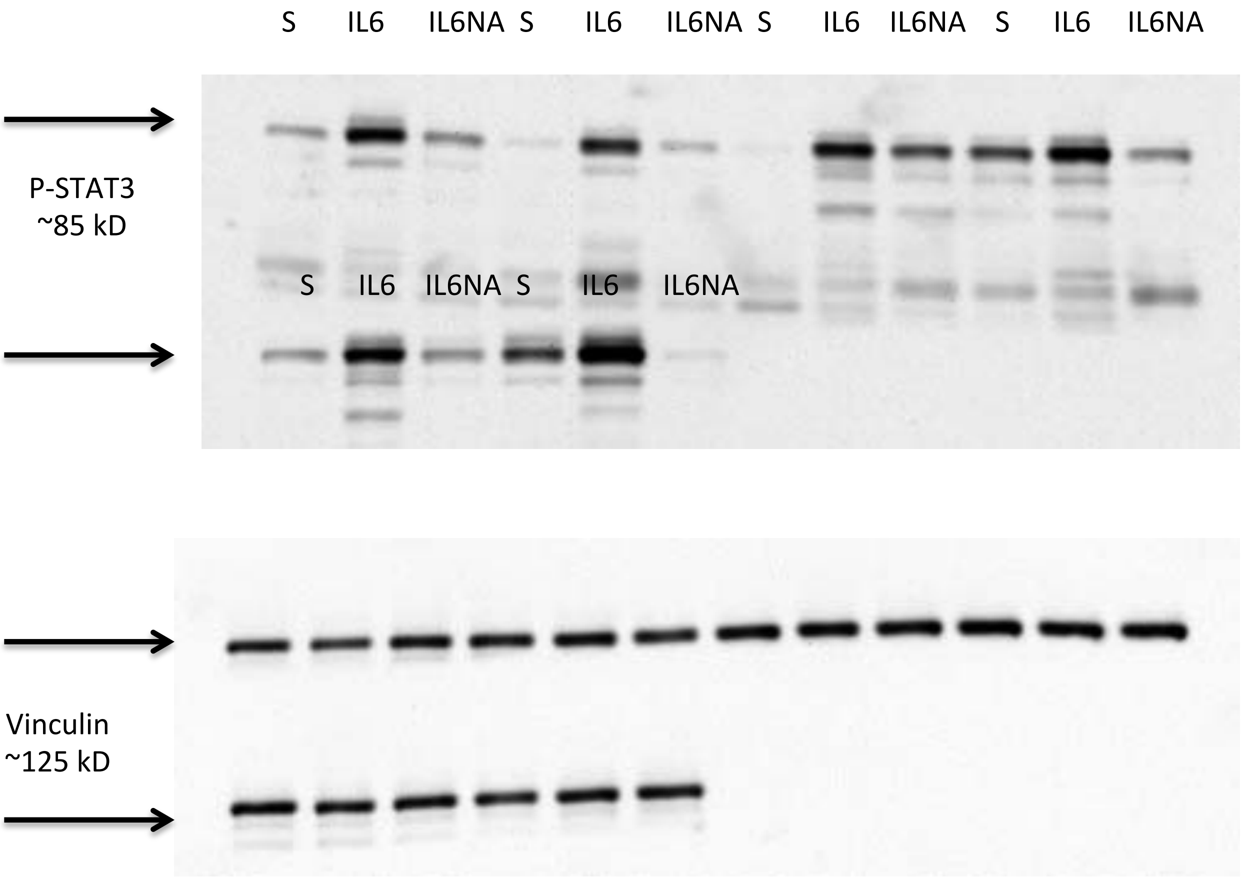

S=saline
IL6=interleukin6
IL6Na=
interleukin6+IL6
neutralizing
antibody

Note: the same order of loading was completed for p-STAT3, STAT3 and the corresponding vinculin blots. Gels were cut and the same proteins were transferred onto the same membrane.

Liver

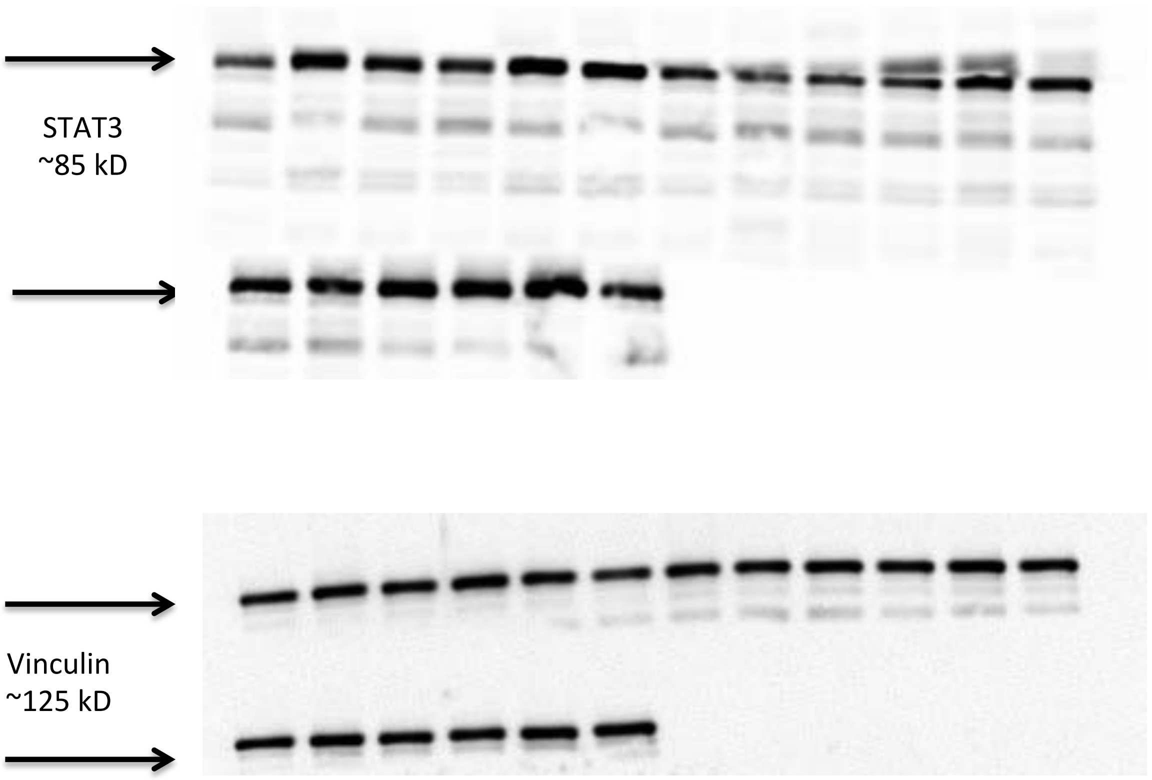

Supplement: Supplementary file 1 — Supplementary Information [file 41598_2018_19260_MOESM1_ESM.pdf]
